# Magnetic Hyperthermia and Oxidative Damage to DNA of Human Hepatocarcinoma Cells

**DOI:** 10.3390/ijms18050939

**Published:** 2017-04-29

**Authors:** Filippo Cellai, Armelle Munnia, Jessica Viti, Saer Doumett, Costanza Ravagli, Elisabetta Ceni, Tommaso Mello, Simone Polvani, Roger W. Giese, Giovanni Baldi, Andrea Galli, Marco E. M. Peluso

**Affiliations:** 1 Cancer Risk Factor Branch, Regional Cancer Prevention Laboratory, ISPO-Cancer Research and Prevention Institute, Florence 50139, Italy; f.cellai@ispo.toscana.it (F.C.); a.munnia@ispo.toscana.it (A.M.); jessicaviti@alice.it (J.V.); 2 Nanobiotechnology Department, Colorobbia Consulting-Cericol, Sovigliana, Vinci 50053, Italy; doumetts@colorobbia.it (S.D.); ravaglic@colorobbia.it (C.R.); baldig@colorobbia.it (G.B.); 3 Department of Experimental and Clinical Biomedical Sciences, University of Florence, Florence 50139, Italy; e.ceni@dfc.unifi.it (E.C.); tommaso.mello@unifi.it (T.M.); simone.polvani@unifi.it (S.P.); a.galli@dfc.unifi.it (A.G.); 4 Department of Pharmaceutical Sciences in the Bouve College of Health Sciences, Barnett Institute, Northeastern University, Boston, MA 02115, USA; r.giese@neu.edu

**Keywords:** magnetic therapy, nanotoxicity, M_1_dG, 8-oxodG, human hepatocarcinoma cells

## Abstract

Nanotechnology is addressing major urgent needs for cancer treatment. We conducted a study to compare the frequency of 3-(2-deoxy-β-d-erythro-pentafuranosyl)pyrimido[1,2-α]purin-10(3*H*)-one deoxyguanosine (M_1_dG) and 8-oxo-7,8-dihydro-2′-deoxyguanosine (8-oxodG) adducts, biomarkers of oxidative stress and/or lipid peroxidation, on human hepatocarcinoma HepG2 cells exposed to increasing levels of Fe_3_O_4_-nanoparticles (NPs) versus untreated cells at different lengths of incubations, and in the presence of increasing exposures to an alternating magnetic field (AMF) of 186 kHz using ^32^P-postlabeling. The levels of oxidative damage tended to increase significantly after ≥24 h of incubations compared to controls. The oxidative DNA damage tended to reach a steady-state after treatment with 60 μg/mL of Fe_3_O_4_-NPs. Significant dose–response relationships were observed. A greater adduct production was observed after magnetic hyperthermia, with the highest amounts of oxidative lesions after 40 min exposure to AMF. The effects of magnetic hyperthermia were significantly increased with exposure and incubation times. Most important, the levels of oxidative lesions in AMF exposed NP treated cells were up to 20-fold greater relative to those observed in nonexposed NP treated cells. Generation of oxidative lesions may be a mechanism by which magnetic hyperthermia induces cancer cell death.

## 1. Introduction

The use of nanotechnology in medicine is of interest for cancer diagnosis and treatment. In regards to treatment, a number of substances are currently under investigation for drug delivery, varying from liposomes to various polymers and magnetic metals [1,2,3,4]. In some cases, the characteristic properties of nanoparticles (NPs) result in their multifunctional potential for simultaneous early detection and treatment of malignant cell growth, such as those of Fe_3_O_4_-NPs [5,6,7]. For example, magnetic hyperthermia is a technique that utilizes the administration of Fe_3_O_4_-NPs into tumor sites and the exposure to alternating magnetic field (AMF) to kill cancer cells by heating. NP properties have been associated with a number of toxic effects, such as chromosomal aberrations, DNA strand breakage, oxidative damage to DNA and mutations [8,9]. Use of NPs along with AMF has been reported to enhance genotoxicity via oxidative stress [10]. In the current study, we focus on the type of genotoxicity in more detail.

A number of assays have been used for the detection of NP-related oxidative damaged to DNA, especially of 8-oxo-7,8-dihydro-2′-deoxyguanosine (8-oxodG), in various experimental cell lines [11]. The interest in 8-oxodG is due to the fact that it is a widely abundant oxidative lesion. Nevertheless, other kinds of oxidative lesions have also been identified in DNA, including the exocyclic 3-(2-deoxy-β-d-erythropentafuranosyl)pyrimido[1,2-α]purin-10(3*H*)-one–deoxyguanosine (M_1_dG) adducts [12], that may be applied to nanotoxicology testing. In particular, the exposure to Fe_3_O_4_-NPs has been associated with enhanced oxidative stress [13]. This overproduction of reactive oxygen species (ROS) may induce lipid peroxidation (LPO) as well as M_1_dG and 8-oxodG [12]. LPO is a process of oxidation of polyunsaturated fatty acids due to the presence of several double bonds in their structure and it involves production of peroxides, ROS, and other reactive species, such as malondialdehyde (MDA). MDA is a reactive LPO by-product, which interacts with DNA forming M_1_dG adducts [14]. Both M_1_dG and 8-oxodG, if not repaired, may induce mutations, such as base pair and frameshift mutations [15] or G:C to T:A transversions [16]. Furthermore, one of the more critical sites on DNA that can be damaged by oxidative damage is the *tumor suppressor P53* [17], a gene involved in cell cycle arrest, DNA repair, senescence, and apoptosis [18]. Early investigations have also indicated that great amounts of both M_1_dG and/or 8-oxodG adducts are associated to cancer development and tumor progression [17,19,20,21,22], therefore these biomarkers are considered worthy indicators for carcinogenesis or mutagenesis events.

Herein, we report a toxicology study aimed at comparing the production of both exocyclic M_1_dG and 8-oxodG adducts, biomarkers of oxidative stress and/or LPO [23], in human hepatocarcinoma HepG2 cells exposed to Fe_3_O_4_-NPs of 50 nm-size in presence or absence of increasing exposures to AMF and prolonged incubations compared to control cells. The levels of exocyclic M_1_dG and 8-oxodG adducts were examined by ^32^P-postlabeling [24,25,26]. Our aim was to investigate the mechanisms underlying the genotoxic effects caused by magnetic hyperthermia. Considering the technical development and the current and future use of this therapy in cancer treatment [27], an improved understanding of the mechanisms of action of magnetic hyperthermia is important.

## 2. Results

### 2.1. Fe_3_O_4_-Nanoparticles and Exocyclic M_1_dG Adducts

One of the purpose of the present study was to evaluate the generation of exocyclic M_1_dG adducts induced by the treatment with increasing amounts of Fe_3_O_4_-NPs, ranging from 30 to 60–90 μg/mL, at different incubation times, from 1 to 48 h. As shown in Table 1, no effects were observed at each concentration for dosages at 2 h of incubations. However, the production of exocyclic M_1_dG adducts per 10^6^ normal nucleotides was increased, up to about two-fold, in HepG2 cells which were treated with NP dosages for incubation times of ≥24 h compared to untreated samples. In our model, the difference between M_1_dG adducts of cells exposed to increasing amounts of Fe_3_O_4_-NPs and basal adduct levels of control cells, i.e., 0.4 ± 0.1 (SE), was generally significant, all *p* < 0.05, after ≥24 h of incubation times, with exception of sample exposed to 30 μg/mL of NPs for 24 h (*p* = 0.057). Table 1 shows that the highest levels of M_1_dG, i.e., 0.9 ± 0.1 (SE), were found in HepG2 cells treated with 60 μg/mL of Fe_3_O_4_-NPs. At higher dosages (90 μg/mL), the treatment was not associated with further adduct production, possibly due to the onset of excessive citoxicity. A significant dose–response relationship was found, with a *p*-value for the trend <0.001. The levels of NP-related M_1_dG adducts were then compared with those caused by the xanthine/xanthine oxidase system, a ROS-generating system [28]. The frequency of exocyclic M_1_dG adducts in HepG2 cells treated with Fe_3_O_4_-NPs was about three-fold lower compared to that induced by the ROS-generating system (Table 1).

### 2.2. Fe_3_O_4_-Nanoparticles and 8-oxodG Adducts 

We next investigated the generation of 8-oxodG in our experimental model. Table 1 reports that there were no effects at each NP concentrations with ≤120 min incubations, whereas adduct production was significantly increased, up to six-fold, after ≥24 of incubations compared to untreated cells. The highest levels of 8-oxodG per 10^6^ normal nucleotides, were found after treatment with 60–90 μg/mL of Fe_3_O_4_-NPs for period of incubation of ≥24, 18.4 ± 2.8 (SE), and 19.4 ± 1.4 (SE), respectively. The difference of 8-oxodG among HepG2 cells treated with various concentrations of Fe_3_O_4_-NPs relative to basal adduct levels, i.e., 3.2 ± 0.2 (SE), was always significant, all *p* < 0.05, after ≥24 h of incubations. The mean amounts of 8-oxodG detected in control cells (3.1–3.2 8-oxodG per 10^6^ normal nucleotides) was in the range of 1.0 and 5.0 per 10^6^ normal dG, which is typically observed in cultured cells [29]. As shown in Table 1, the association between 8-oxodG and NP levels was linear at low dosages, but tended to reach a steady-state at higher exposures. A significant dose–response relationship was observed, *p*-value for the trend <0.001. The amounts of NP-related 8-oxodG were then compared with those detected after treatment with the xanthine/xanthine oxidase system. Table 1 reports that the generation of 8-oxodG was about two-fold higher in HepG2 cells treated with the Fe_3_O_4_-NPs compared to those exposed to the ROS-generating system.

### 2.3. Magnetic Nanoparticles, Alternating Magnetic Field and Exocyclic M_1_dG Adducts

We also evaluated the genotoxicity of Fe_3_O_4_-NPs (90 μg/mL) after increasing exposures, i.e., 20–40 min, to an AMF applied at 186 kHz frequency. Table 2 shows that a significant production of exocyclic M_1_dG adducts, up to a 30-fold increment, was detected in treated cells after AMF exposures compared to controls. The genotoxic effect increased with both exposure and incubation times, with *p*-value for the trend <0.001. The highest value of the exocyclic M_1_dG adducts of 15.9 ± 6.7 (SE) was detected in HepG2 cells containing 90 μg/mL of Fe_3_O_4_-NPs after 20 min of AMF exposure and 48 h of incubation. The difference of M_1_dG was significant, all *p* < 0.05. Table 2 indicates that the levels of exocyclic M_1_dG adducts were also higher, up to 20-fold, in NP treated cells after AMF exposures relative to nonexposed NP treated cells, all *p* < 0.05.

### 2.4. Magnetic Nanoparticles, Alternating Magnetic Field and 8-oxodG Adducts

We investigated the association between the production of 8-oxodG in 90 μg/mL Fe_3_O_4_-NP treated cells after exposure (20 or 40 min) to an AMF applied at 186 kHz. As reported in Table 2, the generation of 8-oxodG was significantly increased, up to 25-fold, in treated cells after AMF exposure compared to controls. The effect was associated to both exposure and incubation, with *p*-value for the trend <0.001. The highest value of 8-oxodG of 79.3 ± 1.1 (SE) was detected in treated cells that were exposed to AMF for 40 min and incubated for 48 h. Table 2 shows that the levels of 8-oxodG were also higher, up to a five-fold increase, in treated cells after AMF exposure relative to nonexposed NP treated cells, all *p* < 0.05.

**Figure 1:**
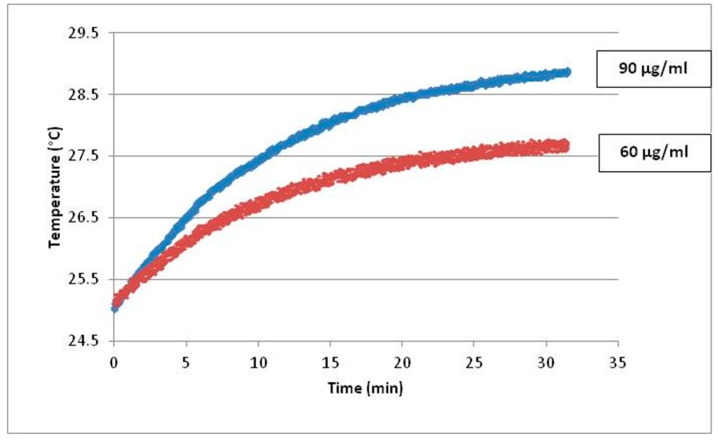
Kinetics of temperature increase versus exposure time for block polymer coated Fe_3_O_4_-NPs 90 µg/mL (blue curve) and 60 µg/mL (red curve).

Since heating curves cannot be directly recorded during the experimental sessions because of sample sterility, kinetic pathways of temperature increase were recorded in solutions of Fe_3_O_4_-NPs containing the same amount of particles as the samples described in the manuscript (Figure 1). Temperature rise of the dispersion during AMF exposure was measured by an optical fiber probe (CEAM Vr18CR-PC) and it was recorded every second during the experimental measurements. The samples were inserted in a homemade polyethylene sample holder, surrounded by glass wool for thermal isolation, and then in the copper coil. The sample mass (*m* = 0*–*3 g) was chosen to minimize the effects of magnetic field inhomogeneity inside the sample.

### 2.5. Fe_3_O_4_-Nanoparticle Internalization

**Figure 2:**
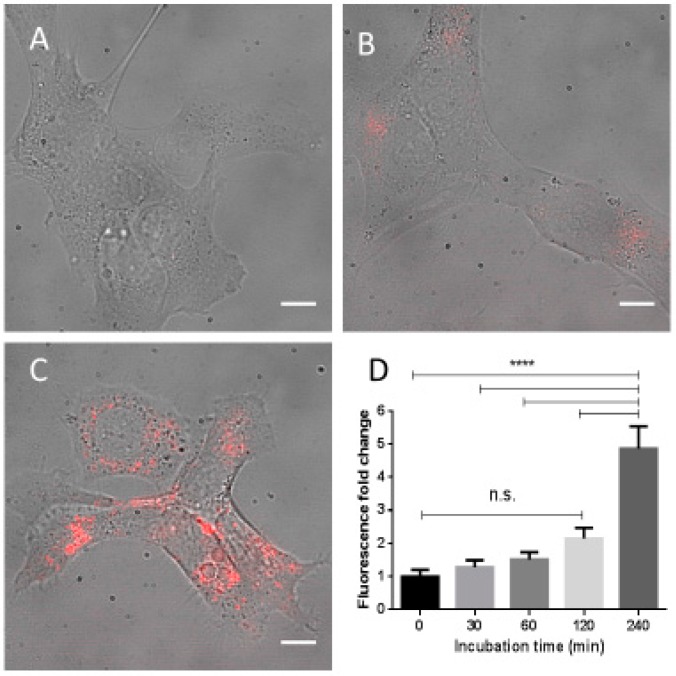
Human hepatocarcinoma (HepG2) cells incubated with 90 μg/mL Fe_3_O_4_-nanoparticles conjugated with fluorescent dye Dylight650. The images of treated cells at selected incubation times are shown: 0 min (**A**); 120 min (**B**); and 240 min (**C**). The increments in fluorescence intensity at each incubation times, mean ± standard error (SE), are reported in the insert (**D**). Scale bar: 10 µm; **** *p* < 0.0001; n.s. = not significant.

The uptake of Fe_3_O_4_-NPs conjugated with fluorescent dye Dylight650 was monitored by fluorescent microscopy. NP fluorescence inside cells was measured with background subtraction. As shown in Figure 2A–C, the NPs were internalized at the concentration of 90 μg/mL by HepG2 cells in sufficient quantity to be detected after 240 min (Figure 2C). A significant increase in fluorescence intensity was indeed observed only at that incubation time, *p* < 0.0001 (Figure 2D). The lack of genotoxic effects in Fe_3_O_4_-NPs treated cells at lower incubation time is apparently due to a lack of significant cellular penetration of NPs during this initial period.

## 3. Discussion

The potential therapeutic benefits of Fe_3_O_4_-NPs [30] must consider their potential for off-target toxicity [8,9,31,32,33]. Fe_3_O_4_-NPs, if undirected, are mainly sequestered in liver tissue for elimination. Hence, we studied human hepatocarcinoma cells for NP-related toxicity. In particular, we investigated the pro-oxidant properties of Fe_3_O_4_-NPs in towards DNA. We asked whether experimental cells treated with increasing NP concentrations experienced genotoxic effects at various times of incubation. A further purpose of the present study was to investigate the generation of oxidative DNA lesions in Fe_3_O_4_-NP treated cells after AMF exposures. Two different biomarkers, i.e., exocyclic M_1_dG and 8-oxodG adducts, of oxidative stress and/or LPO [23], were measured by ^32^P-postlabeling.

**Figure 3:**
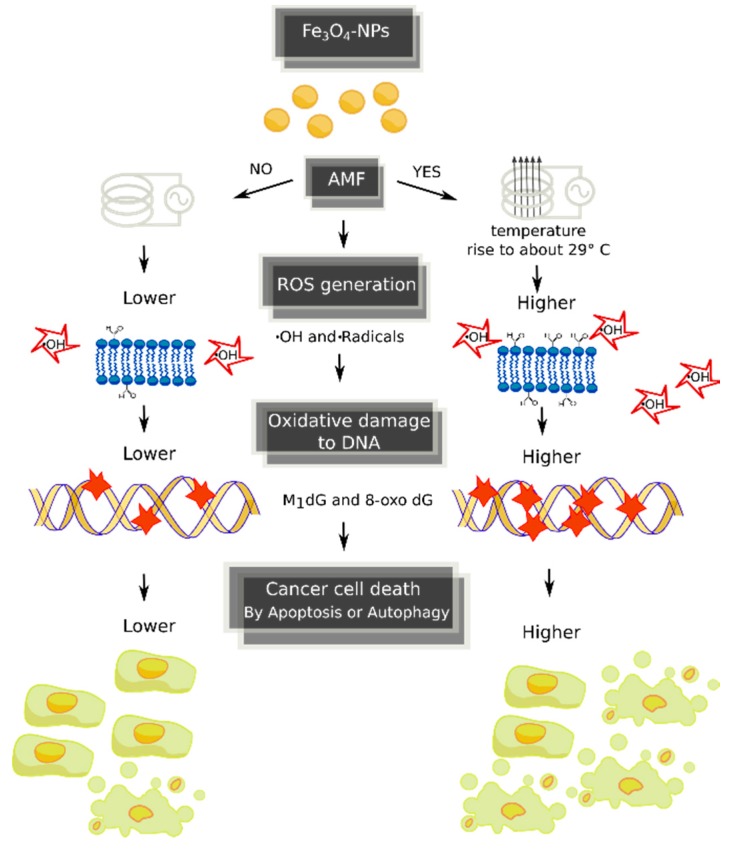
Potential mechanisms underlying the genotoxic effects caused by magnetic hyperthermia. AMF, alternating magnetic field; ROS, reactive oxygen species.

High dosages of Fe_3_O_4_-NPs are expected to significantly increase cellular ROS and the production of oxidative damage to DNA [11]. This was found in our model, where the administration of increased NP concentrations (30, 60 or 90 μg/mL) was significantly associated with higher levels of both exocyclic M_1_dG and 8-oxodG adducts in HepG2 cells after ≥24 h of incubation in comparison to untreated cells. No genotoxic effects were detected by each concentration dosages at ≤120 min of incubations, possibly due to a lack of penetration of NPs into cellular membrane of HepG2 cells during this initial period, as shown by fluorescent microscopy (Figure 2). We found a significant dose–response relationship between oxidized DNA lesions and Fe_3_O_4_-NPs. Nevertheless, while the associations with adduct production tended to be linear at low exposure, it was sublinear at high dosages, perhaps due to excessive cytotoxicity. We assume in this study that Fe_3_O_4_-NPs act as a pro-oxidant by causing iron-dependent Fenton reactions that generate first ^.^OH and, then, ^.^C radicals, through LPO induction, which are important intermediates that lead to the formation of oxidative DNA lesions. Consequently, the addition of Fe_3_O_4_-NPs to HepG2 cells increases the oxidation of genomic DNA, measured as promutagenic lesions. The toxic action of magnetic therapy may be mediated by several mechanisms, as shown in Figure 3.

Our findings are in keeping with a series of earlier reports which have shown adverse effects associated with the exposure to Fe_3_O_4_-NPs in mammalian liver cells [11]. For example, Lin and coworkers [34] analyzed the toxicity of Fe_3_O_4_-NPs on human hepatocytes after 24 h of incubation using various assays including the comet assay and other biomarkers of oxidative stress, such as glutathione peroxidase, superoxide dismutase and MDA. In that study, the treatment with NPs was associated with both nuclear condensation and chromosomal DNA fragmentation. Significant reductions of cellular levels of glutathione peroxidase and superoxide dismutase levels as well as increased MDA were also observed. Sadeghi and coworkers [35] analyzed the effects of Fe_3_O_4_-NP exposures (75–100 µg/mL) in HepG2 cells after 12–24 h of treatment. Higher oxidative damage to DNA that was both concentration and time dependent was detected. In another study, Ma and coworkers [36] analyzed the effects of increasing dosages up to 40 mg/kg of Fe_3_O_4_-NPs on mice after seven days of treatment. Significant increments of both 8-oxodG and DNA-protein crosslinks were observed in liver tissue.

Magnetic hyperthermia is a technique based on the use of Fe_3_O_4_-NPs to remotely induce local heat in cancer cells when AMF is applied. Increased production of oxidative stress has been associated with this therapeutic approach [10,37]. We evaluated whether DNA-damaging effects induced by Fe_3_O_4_-NPs (90 µg/mL) are intensified by increasing exposures to AMF. Our most striking result shows that higher exposures to AMF induce a significant production of both exocyclic M_1_dG and 8-oxodG adducts compared to controls cells. Thus, one important question is whether the increasing induction of DNA-modifications is correlated to a rising temperature. This is important, since as known chemical reactions can be promoted by higher temperatures. The effects, shown in the data reported in Figure 1, showed that oxidative lesions were induced with a temperature rise of 4 °C, but it is reasonable that the punctual heating of each single particle under AMF reaches even higher temperature that, to date, is not possible to be quantified. The genotoxic effects were also significantly associated with incubation times (24–48 h). The levels of oxidative DNA lesions in AMF exposed treated cells were also significantly higher in respect to nonexposed NP treated cells. Specifically, the levels of exocyclic M_1_dG adducts in AMF exposed NP treated cells are up to 95% higher when compared to nonexposed NP treated cells. The amounts of 8-oxodG are up to 73% greater in respect to nonexposed controls. Nevertheless, the levels of M_1_dG in AMF exposed NP treated HepG2 cells (30-fold increment vs. nonexposed control cells) was lower than that detected in A549 and BEAS-2B lung epithelial cells exposed to 10 µM of benzo(a)pyrene (54-fold increment vs. control cells) [38]. Our findings are in agreement with Shaw and coworkers [37], who observed enhancement of genotoxic effects by Fe_3_O_4_-NPs in cancer cells after magnetic flux using the Comet assay. In that study, magnetic field exposure increased the levels of DNA double and single strand breaks in cancer cells. 

On the one hand, Fe_3_O_4_-NPs may absorb the energy from AMF and convert it into heat primarily through Brownian and Neel relaxations, when exposed to AMF [10]. The heat in turn could simply increase reaction rates for existing ROS. On the other hand, magnetic therapy could increase formation of ROS such as hydroxyl radicals at the surface of Fe_3_O_4_-NPs [39]. Increased levels of ferrous ions might form and react with H_2_O_2_ produced by mitochondria, inducing ROS generation through the Fenton reaction [40,41], which causes various kind of oxidative damage to DNA, such as exocyclic M_1_dG and 8-oxodG adducts. Alternatively, ROS induction may be attributed to surface coating-cell interactions. Toxic effects of Fe_3_O_4_-NPs may partially derive from chemical reactions between coating and cell components during phagocytosis [42]. In support to this hypothesis, we showed an association between oxidative burst of activated cells and M_1_dG generation [43,44].

The generation of oxidative DNA lesions, such as those caused from Fe_3_O_4_-NPs [45], has intensified interest in nanotoxicology because they are considered one way to induce of authophagy and apoptosis cell death of cancer cells [46]. When DNA is severely damaged or unrepaired, cells may remain quiescent and activate pro-survival mechanisms or undergo cell death. Once induced by DNA injury, autophagy may delay apoptosis in response to genetic damage by sustaining the mechanisms necessary for DNA repair, or may contribute to cell death when DNA is unrepaired and apoptosis is defective.

## 4. Material and Methods

### 4.1. Fe_3_O_4_-Nanoparticles, Cell Culture and Cell Treatments in Presence or Absence of Alternating Magnetic Field

**Figure 4:**
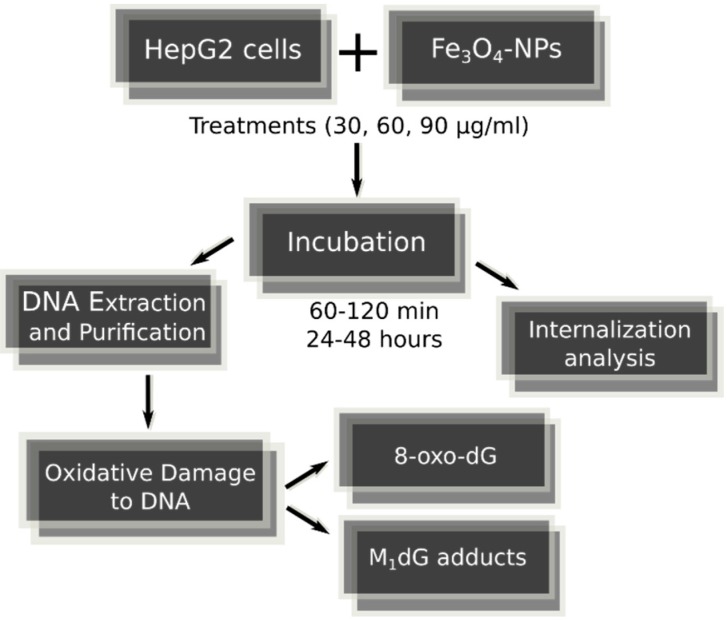
Strategy protocol of analytic research aimed to evaluate genotoxic activity of Fe_3_O_4_-nanoparticles with increasing dosages and incubation times in HepG2 cells.

**Figure 5:**
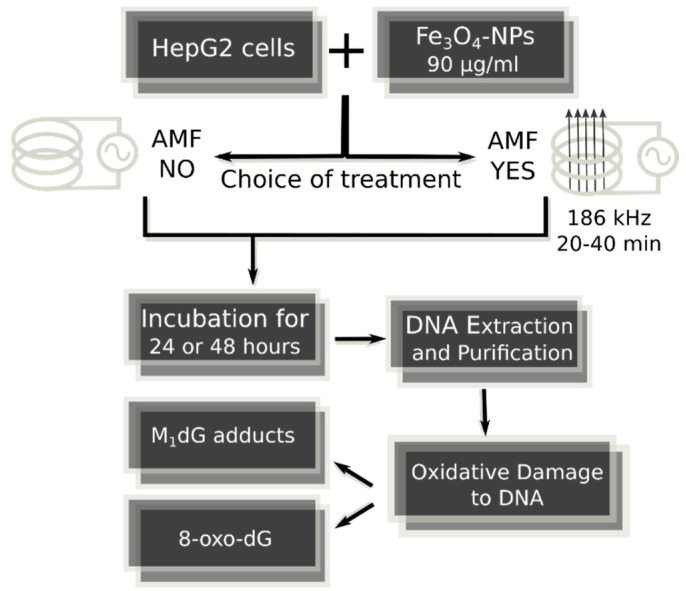
Strategy protocol of analytic research aimed to examine the genotoxic effects of Fe_3_O_4_-nanoparticles at select dosage and incubation times in presence or absence of magnetic therapy in HepG2 cells.

The 50-nm hydrodynamic diameter size (by DLS measurements) block polymer coated Fe_3_O_4_-NPs of 3 mg/mL concentration were obtained from Colorobbia Consulting-Cericol, Italy. HepG2 cells were routinely cultivated under standard conditions. Cells were incubated in a humidified controlled atmosphere with a 95% to 5% ratio of air/CO_2_, at 37 °C, with medium that was changed every 3 days. Trypsinized cells at 30–40% confluence were exposed to increasing levels of Fe_3_O_4_-NPs that were dispersed in cell culture medium to obtain the final concentrations of 30, 60 or 90 μg/mL. Increasing lengths of incubations were used, i.e., from 60–120 min to 24–48 h. The entire and general image of strategy of the present research is shown in Figure 4 and Figure 5.

Specifically, the NP concentrations were comparable to those utilized in the studies of Kham and coworkers [47], and Alarifi and coworkers [48], who found increased toxic effects caused from the treatments with Fe_3_O_4_-NPs of comparable diameter size. Subsequently, the hepatocarcinoma cells, which were treated with 90 µg/mL of NPs, were exposed to 25 kA/m AMF at a frequency of 186 kHz with a copper coil of 3 turns and Ø = 100 mm (Celes MP 6/400) for 20 or 40 min exposure times, and then returned to the incubator for 24 or 48 h of incubation. Cells not exposed to NPs served as negative control in each experiment. As positive internal control, experimental cells were treated with 0.2 mM xanthine plus 1.0 or 5.0 mU xanthine oxidase, a system capable of generating singlet oxygen and hydrogen peroxide [28], and incubated for 24–48 h.

### 4.2. Preparation of M_1_dG Reference Adduct Standard

A reference adduct standard was prepared: calf-thymus-DNA was treated with 10 mM MDA (ICN Biomedicals, Irvine, CA, USA), as previously reported [49]. MDA treated calf-thymus-DNA was diluted with untreated DNA to obtain decreasing levels of the reference adduct standard to generate a calibration curve.

### 4.3. Mass Spectrometry Analysis

The levels of exocyclic DNA adducts in MDA treated calf-thymus-DNA sample were analyzed by mass spectrometry (Voyager DE STR from Applied Biosystems, Framingham, MA, USA), as reported elsewhere [50].

### 4.4. Reference M_1_dG Standard by ^32^P-Postlabeling and Mass Spectrometry

The levels of the exocyclic M_1_dG adducts were 5.0 ± 0.6 per 10^6^ normal nucleotides in MDA treated calf-thymus-DNA using ^32^P-postlabeling [24]. The presence of exocyclic M_1_dG adducts in this sample was confirmed by matrix-assisted laser desorption/ionization time-of-flight-mass-spectrometry (MALDI-TOF-MS). A calibration curve was set up by diluting this sample with untreated calf-thymus-DNA and measuring the decreasing amount of M_1_dG, *R*^2^ = 0.99.

### 4.5. DNA Isolation and Hydrolysis

DNA from treated and untreated cells was extracted and purified from lysed cells using a method that requires digestion with ribonuclease A, ribonuclease T_1_ and proteinase K treatment and extraction with saturated phenol, phenol/chloroform/isoamyl alcohol (25:24:1), chloroform/isoamyl alcohol (24:1) and ethanol precipitation [51]. DNA concentration and purity were determined using a spectrophotometer. Coded DNA samples were subsequently stored at −80 °C until laboratory analyses. Before adduct determination, DNA (2–5 μg) was hydrolyzed by incubation with micrococcal nuclease (21.45 mU/μL) and spleen phosphodiesterase (6.0 mU/μL) (Sigma Aldrich, St. Louis, MO, USA and Worthington, NJ, USA) in 5.0 mM Na succinate, 2.5 mM calcium chloride, pH 6.0 at 37 °C for 4.5 h [52,53].

### 4.6. Exocyclic M_1_dG Adduct Analysis

The generation of M_1_dG adducts was analyzed by ^32^P-postlabeling [24]. Hydrolyzed samples were incubated with nuclease P1 (0.1 U/μL) in 46.6 mM sodium acetate, pH 5.0, and 0.24 mM ZnCl_2_ at 37 °C for 30 min. After nuclease P1 treatment, 1.8 μL of 0.16 mM Tris base was added to the sample. The nuclease P1-resistant nucleotides were incubated with 7–25 μCi of carrier-free [γ-^32^P]ATP (3000 Ci/mM) and polynucleotide kinase T_4_ (0.75 U/μL) to generate ^32^P-labeled DNA adducts in bicine buffer, 20 mM bicine, 10 mM MgCl_2_, 10 mM dithiotreithol, 0.5 mM spermidine, pH 9.0, at 37 °C for 30 min. The generation of M_1_dG adducts in Fe_3_O_4_-NPs treated hepatocarcinoma cells and control cells were measured using a modified version of ^32^P-postlabeling [38]. Labeled samples were applied on polyethyleneimine cellulose thin-layer chromatography plates (Macherey-Nagel, Postfach, Germany). The chromatographic analysis of M_1_dG adducts was performed using a low-urea solvent system known to be effective for the detection of low molecular weight and highly polar DNA adducts: 0.35 M MgCl_2_ for the preparatory chromatography; and 2.1 M lithium formate, 3.75 M urea pH 3.75 and 0.24 M sodium phosphate, 2.4 M urea pH 6.4 for the two-dimensional chromatography.

Detection and quantification of modified and normal nucleotides, i.e., diluted samples that were not treated with nuclease P1, was performed by storage phosphor imaging techniques employing intensifying screens from Molecular Dynamics (Sunnyvale, CA, USA). The intensifying screens were scanned using a Typhoon 9210 (Amersham, UK). Software used to process the data was ImageQuant from Molecular Dynamics. After background subtraction, M_1_dG adducts were expressed as relative adduct labeling (RAL) = pixel in adducted nucleotides/pixel in normal nucleotides. M_1_dG levels were corrected across experiments based on the recovery of reference M_1_dG adduct standard.

### 4.7. 8-oxodG Adduct Analysis

Digest DNA was diluted with ultrapure water to 20 ng/μL. Diluted DNA digest was incubated with 10 μCi of carrier-free [γ-^32^P]ATP (3000 Ci/mM) (Amersham, UK) and 2 U of polynucleotide kinase T4 (10 U/μL) (Roche Diagnostics, Indianapolis, IN, USA) to generate ^32^P-labeled adducts in the reaction buffer (10×) at 37 °C for 45 min [21,52]. ^32^P-labeled samples were treated with 1.2 U of nuclease P1 (1.9 U/μL) (Sigma Aldrich, St. Louis, MO, USA) in 62.5 mM sodium acetate, pH 5.0, and 0.27 mM ZnCl_2_ at 37 °C for 60 min (final volume 10 μL) [25,26,53]. ^32^P-labeled samples were applied on polyethyleneimine cellulose thin-layer chromatography plates (Macherey-Nagel, Postfach, Germany). The chromatographic analysis of 8-oxodG, a biomarker of oxidative stress and cancer risk [54], has been performed using a chromatographic system known to be effective for the detection and quantitative analysis of such oxidative lesions [25,26,55,56,57,58]. As previously reported [25], this procedure specifically detects 8-oxodG, which is the only adduct retained in chromatograms developed in acidic medium. Specifically, an aliquot (2.5 μL) of the labeled solution was applied to the origin of a chromatogram and developed overnight onto a 3 cm-long Whatman 1 paper wick with 1.5 M formic acid for the one-dimensional chromatography. For the two-dimensional chromatography, chromatographic plates were developed at the right angle to the previous development with 0.6 M ammonium formate, pH 6.0. Detection and quantification of 8-oxodG was obtained as described above. After background subtraction, the levels of 8-oxodG were expressed such as RAL = screen pixel in 8-oxodG spot/screen pixel in total normal nucleotides [25,26]. The levels of the normal nucleotides were measured as described above. The values measured for the 8-oxodG spot were corrected across experiments based on the recovery of the internal standard, e.g., 8-oxodG (Sigma Aldrich, St. Louis, MO, USA).

### 4.8. Fe_3_O_4_-Nanoparticle Internalization

To evaluate the dynamics of cellular internalization, Fe_3_O_4_-NPs were conjugated with the fluorescent dye DyLight650 and imaged by live-cell microscopy. Briefly, HepG2 cells exposed to 90 μg/mL of Fe_3_O-NPs were cultured in FluoroBrite DMEM media supplemented with 10% FCS, and kept at 37 °C with 5% CO_2_ during image acquisition. Images were acquired through a 63x HCX PLANAPO 1.2NA water-immersion objective, using a Leica AM6000 inverted microscope equipped with a DFC350FX camera. Images were collected before addition of NPs (*t* = 0) to analyze background and autofluorescence of experimental cells, and incubated at different incubation times, i.e., 30, 60, 120, or 240 min. Both transmitted and fluorescence images were collected. Quantification of intracellular fluorescence was performed using Fiji software [59] by manually drawing regions-of-interest (ROI) around cells in the brightfield channel and measuring the mean intensity fluorescence in each region. Mean background fluorescence was measured in each microscopic field (by drawing ROI not-containing cells) and subtracted from cellular fluorescence values. Fluorescence intensity values were normalized by the average fluorescence intensity at *t* = 0.

### 4.9. Statistical Analysis

The levels of oxidative DNA lesions were expressed as adducted nucleotides per 10^6^ normal nucleotides. Adduct data were log transformed to stabilize the variance and normalize the distribution. Baseline characteristics between two groups were compared using independent t-test or Mann–Whitney *U* test for continues variables and chi square test for categorical variables. All statistical tests were two-sided and *p* < 0.05 was considered to be statistically significant. The data were analyzed using SPSS 13.0 (IBM SPSS Statistics, New York, NY, USA).

## 5. Conclusions

The greater production of Fe_3_O_4_-NPs-related oxidative DNA lesions after exposures to AMF may be one of the mechanisms by which magnetic therapy kills cancer cells. Concerning cancer cell death specifically, the hypothesis that increased levels of oxidative damage to DNA during magnetic therapy might contribute to kill cancer cells upon greater induction of apoptosis and autophagy seems to be fair enough. The generation of oxidative DNA lesions might be a further parameter that has to be maximized to optimize the action of this therapeutic approach.

## Figures and Tables

**Table 1:** Mean levels of 3-(2-deoxy-β-d-erythro-pentafuranosyl)pyrimido[1,2-α]purin-10(3*H*)-one deoxyguanosine (M_1_dG) adducts and 8-hydroxy-2′-deoxyguanosine (8-oxodG) per 10^6^ normal nucleotides in HepG2 cells after treatment with 30, 60 or 90 µg/mL of Fe_3_O_4_-nanoparticles and prolonged incubation times compared to control cells. Adduct levels caused by a free radical-generating system in our model are reported, as positive internal control.

Exocyclic M_1_dG and 8-oxodG Lesions					
	*N*	M_1_dG ± SE	*p*-Value	8-oxodG ± SE	*p*-Value
Adduct background					
Control cells					
Incubation times					
24 h ^a^	10	0.4 ± 0.1		3.2 ± 0.2	
48 h ^a^	10	0.4 ± 0.1		3.1 ± 0.2	
Adduct levels of magnetic nanoparticle treated cells					
Treated cells					
30 µg/mL					
Incubation times					
24 h	10	0.5 ± 0.1	0.057 ^b^	11.2 ± 0.8	<0.05 ^b^
48 h	10	0.6 ± 0.1	<0.05 ^b^	14.7 ± 0.4	<0.05 ^b^
60 µg/mL					
Incubation times					
24 h	10	0.8 ± 0.1	<0.05 ^b^	18.4 ± 2.8	<0.05 ^b^
48 h	10	0.9 ± 0.1	<0.05 ^b^	19.4 ± 1.4	<0.05 ^b^
90 µg/mL					
Incubation times					
24 h	10	0.7 ± 0.1	<0.05 ^b^	17.1 ± 1.7	<0.05 ^b^
48 h	10	0.8 ± 0.1	<0.05 ^b^	18.0 ± 1.8	<0.05 ^b^
Adduct levels of positive internal control					
Free radical-generating system					
0.2 mM xanthine/1.0 mU xanthine oxidase					
Incubation times					
24 h	10	1.9 ± 0.1	<0.05 ^b^	4.6 ± 0.2	<0.05 ^b^
48 h	10	2.2 ± 0.1	<0.05 ^b^	5.4 ± 0.2	<0.05 ^b^
0.2 mM xanthine/5.0 mU xanthine oxidase					
Incubation times					
24 h	10	2.9 ± 0.6	<0.05 ^b^	8.3 ± 0.2	<0.05 ^b^
48 h	10	2.7 ± 0.4	<0.05 ^b^	9.3 ± 0.4	<0.05 ^b^

^a^ Reference levels; ^b^ *p*-values vs. appropriated controls.

**Table 2:** Mean levels of 3-(2-deoxy-β-d-erythro-pentafuranosyl)pyrimido[1,2-α]purin-10(3*H*)-one deoxyguanosine (M_1_dG) adducts and 8-hydroxy-2′-deoxyguanosine (8-oxodG) adducts per 10^6^ normal nucleotides in 90 µg/mL Fe_3_O_4_-nanoparticle (NP) treated HepG2 cells in presence or absence of alternating magnetic field (AMF) exposures and prolonged incubation times compared to control cells.

Exocyclic M_1_dG and 8-oxodG Adducts					
	*N*	M_1_dG ± SE	*p*-Value	8-oxodG ± SE	*p*-Value
Adduct background in presence or absence of AMF exposures					
Control cells					
Nonexposed to AMF					
Incubation times					
24 h ^a^	10	0.4 ± 0.1		3.2 ± 0.2	
48 ^a^	10	0.4 ± 0.1		3.1 ± 0.2	
Exposed to AMF (20 min)					
Incubation times					
24 h ^a^	10	0.5 ± 0.1		3.2 ± 0.2	
48 ^a^	10	0.5 ± 0.1		3.3 ± 0.3	
Exposed to AMF (40 min)					
Incubation times					
24 h ^a^	10	0.5 ± 0.1		3.4 ± 0.2	
48 ^a^	10	0.5 ± 0.1		3.9 ± 0.3	
Adduct levels of treated cells in presence or absence of AMF exposures					
NP treated cells					
Nonexposed to AMF					
Incubation times					
24 h ^a^	10	0.7 ± 0.1	<0.05 ^b^	16.7 ± 1.7	<0.05 ^b^
48 ^a^	10	0.8 ± 0.1	<0.05 ^b^	21.2 ± 1.9	<0.05 ^b^
Exposed to AMF (20 min)					
Incubation times					
24 h ^a^	10	4.6 ± 0.5	<0.05 ^b^	40.3 ± 3.3	<0.05 ^b^
48 ^a^	10	6.9 ± 0.6	<0.05 ^b^	69.3 ± 1.4	<0.05 ^b^
Exposed to AMF (40 min)					
Incubation times					
24 h ^a^	10	6.4 ± 1.4	<0.05 ^b^	36.5 ± 4.0	<0.05 ^b^
48 ^a^	10	15.9 ± 6.7	<0.05 ^b^	79.3 ± 1.1	<0.05 ^b^

^a^ Reference levels; ^b^ *p*-values vs. appropriated referent cells.
